# Adhesion Depends on Interfacial Strength: Time and Temperature Effects

**DOI:** 10.1021/acs.langmuir.6c02054

**Published:** 2026-06-18

**Authors:** Shi-Qing Wang, Zehao Fan, Tianji Pang, Zhe Cui

**Affiliations:** School of Polymer Science and Polymer Engineering, The University of Akron, Akron, Ohio 44325, United States

## Abstract

In the conventional description, peeling of an adhesive from a solid substrate is expressed in terms of adhesion energies at adhesive-substrate interfaces. However, this energy-based perspective faced considerable difficulties in explaining why the adhesion energy, also known as peel strength Γ_p_, is many orders of magnitude greater than Dupré’s thermodynamic work of adhesion, Γ_0_. In this study, we present experimental evidence to demonstrate that polymer adhesion is governed by interfacial strength σ_interf_, defined by the pair of adhesive and substrate. A tensile adhesion test is performed to estimate σ_interf_ from adhesion strength σ_adh_ equal to the engineering stress at detachment. This test stretches, at various rates under different temperatures, one end of a ribbon-like specimen with the other end adhered to the same substrate used in peeling tests until adhesive detachment. The dependence of σ_adh_ on applied rate and temperature is found to be the same as that of Γ_p_ from conventional peeling tests on peeling speed *v*_p_ and temperature. Our stress perspective shows that Γ_p_ explicitly correlates with σ_adh_ through a characteristic length scale *P* instead of Γ_0_. Here, *P* is the distance from the peeling front, beyond which the adhesive undergoes little deformation. Since *P* is a much larger length scale than a molecular scale and σ_adh_ directly depends on polymer–substrate interfacial interactions, Γ_p_ explicitly depends on σ_interf_. As activated processes, in both peeling and tensile adhesion tests, interfacial debonding on shorter time scales and lower temperature required higher interfacial stress.

## Graphical abstract

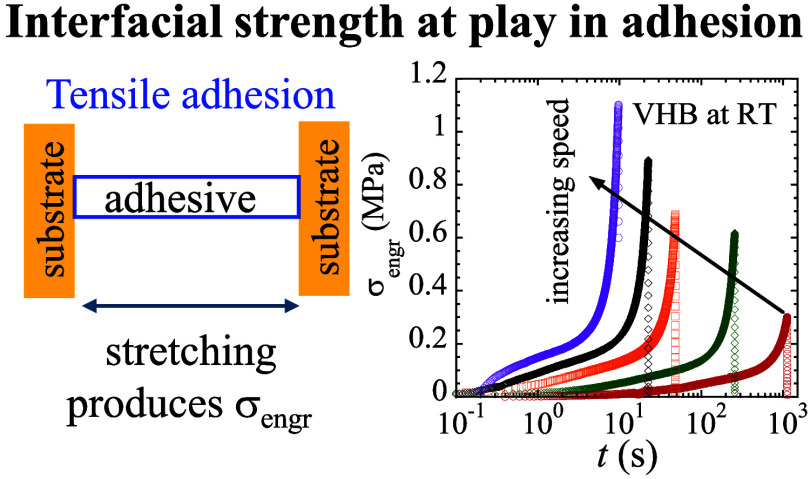

## 1 Introduction

Polymer adhesion^1^ is commonly interpreted within the Dupré work-of-adhesion (Γ_0_) framework,^2–4^ where peel strength Γ_p_ is regarded to reveal adhesion fracture energy. On the other hand, Γ_p_ is orders of magnitude greater than Γ_0_, whose magnitude can be comparable to the surface energy of the adhesive. Specifically, peeling tests^5–24^ [Bibr newref25] are often characterized by Γ_p_ in the following form^25^ as a function of peeling speed *v*_p_ and temperature *T*:

1 $$\Gamma_{p}=\Gamma_{0}\left[1+f_{\mathrm{adh}}\left(v_{p},T\right)\right],,\mathrm{with}\;f_{\mathrm{adh}}\gg 1$$

where the second component *f*_adh_ is supposed to arise from viscoelastic dissipation associated with bulk deformation of the adhesive.^15,16^ In the present study, we suggest, based on new experiments, that polymer adhesion should be understood in a different way, i.e., in terms of interfacial strength σ_interf_ instead of Γ_0_. Peel strength, Γ_p_, as a measure of mechanical resistance against interfacial debonding of the adhesive off the substrate, directly manifests interfacial strength. We suggest that a tensile adhesion test can be carried out to estimate interfacial strength σ_interf_ on different time scales at different temperatures. We will investigate whether Γ_p_ depends on temperature and characteristic time scales, associated with peeling speed *v*_p_ and stretching rate λ̇ in peeling tensile adhesion tests, as σ_interf_ does.

Tensile adhesion and peeling experiments can be carried out to measure adhesion strength σ_adh_ and peel strength Γ_p_. These two quantities are related through a length scale *P* that measures an effective region over which peeling force acts on the interface at the peeling front, beyond which adhesive experiences little deformation. This characteristic length *P* largely depends on interfacial strength, along with thickness *B* and the stretchability of adhesives. Our description of adhesion in terms of Γ_p_/*P* ≈ σ_adh_ offers a stress perspective to the current paradigm that treats adhesion from Griffith’s energy perspective,^26,27^ i.e., in terms of Γ_p_. In this alternative framework Γ_p_ is expressed in terms of normalized force (σ_adh_) required to exceed the interfacial strength, which is time and temperature dependent, along with P.

This paper is organized as follows. In Section 2, we not only describe a rarely adopted experimental protocol to characterize interfacial strength but also present our analysis and methodology. Specifically, we discuss the relationship between probe tests and peeling tests. We show various results from different experiments in Section 3. Specifically, Section 3.1 describes an experimental estimate of interfacial strength in terms of adhesion strength σ_adh_ based on both types of probe tests (tensile adhesion and poker chip), defined as the engineering stress at adhesive failure as a function of probe speed and temperature. Results from peeling tests at different peeling speeds and temperatures are presented in Section 3.2. Using adhesives of different thicknesses (0.02 vs 1 mm), we show that peeling tests are characterized by both peel strength, Γ_p_, and characteristic length scale, *P*. Interfacial strength prescribes peel strength. Thus, peel strength increases with increasing peeling speed and decreasing temperature because overcoming interfacial strength is an activated process, i.e., both time-dependent and temperature-sensitive. When peeling only involves adhesive failure, we conclude in Section 4 that (a) time- and temperature-dependent interfacial debonding explains the phenomenology summarized by eq 1 and (b) characteristics such as time- and temperature-dependent peel strength Γ_p_ from peeling tests and debonding stress σ_adh_ from probe tests merely reflect how interfacial interactions between adhesive and substrate vary on different time scales at different temperatures.

## 2 Experimental and Technical Backgrounds

### 2.1 New Method—Tensile Adhesion for Probe/Tack Tests

To clarify the nature of peeling that involves only adhesive failure, we perform tensile adhesion tests, as depicted in Figure 1a. Upon placing a freshly fractured VHB strip on various substrates including bare glass and steel and after a given amount of time for adsorption, the other end of the strip is fixed onto an Instron clamp while the substrate is held stationary to act like the second clamp in such a tensile test. At different values of stretching rate λ̇ = *V*/*L*_0_, engineering stress σ_engr_ = *F*/*BW* is measured as a function of extension until termination by adhesive failure at the substrate. Taking σ_adh_ to be given by σ_engr_ at debonding in such a tensile adhesion test, we measure σ_adh_ as a function of the time elapsed to the point of interfacial detachment, i.e., *t*_db_ = (λ_adh_ – 1)/λ̇.

**Figure 1:**
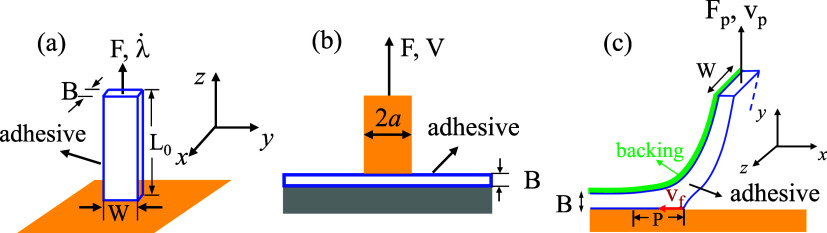
(a) Tensile adhesion test is defined as shown, where the substrate is in contact with adhesive of area *B* × *W*. (b) Poker chip-like configuration, where the layer of adhesive may match the probe size or be a larger sheet. The probe is round-shaped with a diameter *a*. (c) Peeling test for adhesive with thickness *B* fixed on the backing. A force arises from an area of size *P* × *W*, which is maximum at the peeling front.

### 2.2 Materials and Their Preparation

Two commercially available adhesives are used to describe pertinent physics involved in polymer adhesion. They are VHB4910 and Scotch tape, both purchased from Amazon. VHB is in the form of sheets with a thickness of *B* = 1.0 mm. Scotch tape refers to a commercial transparent tape (Scotch Transparent Tape, Cat. 600, 3M) and has a thickness of 0.02 mm for its adhesive layer. Prior to testing, substrates were carefully cleaned following the sequence of hexane, alcohol, and water, and then dried by compressed air. All specimens were handled using tweezers to avoid contamination.

Both probe/tack and peeling tests are based on the free-spinning wheel device shown in Figure 2a, along with an actual photograph of the apparatus in Figure 2b, which was earlier used^5^ by Kaelble. The substrate for tensile adhesion tests is either the surface of the wheel, made of either a borosilicate glass tube with a diameter of ca. 50 mm or steel tubes with a diameter of 48 mm, or steep tube wrapped with plastic films (Mylar and Kapton).

**Figure 2:**
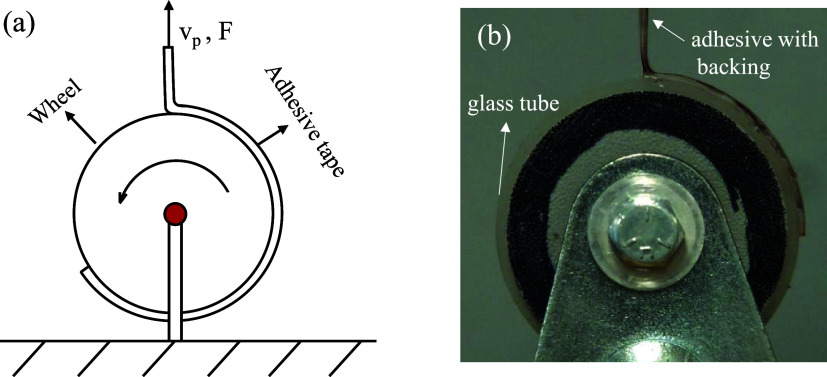
(a) Schematic of the wheel device for 90° peeling that is fixed on the base of the Instron 5969 universal testing instrument equipped with a temperature-controlled environmental chamber. The upper clamp of the Instron holds the specimen to travel upward for all three types of tests depicted in Figure 1a–c. (b) Photo of the actual peeling device.

To prepare specimens for tensile adhesion tests, VHB sheets were cut into rectangular specimens with approximate dimensions of 15 mm × 12.5 mm × 1 mm (length × width × thickness). The strips were sufficiently stretched in uniaxial extension so that a small notch introduced with scissor cutting triggers instant rupture, creating two smooth and flat fracture surfaces to be introduced into contact with the substrate for tensile adhesion tests.

For all poker chip tests, the probe’s surface is made of a circular glass disk with diameters *a* = 3 mm that is fixed onto the upward traveling clamp at various speeds after being brought into contact with a flat stationary VHB sheet with typical dimensions of 110 mm × 70 mm × 1 mm, naturally achievable because VHB is a double-sided adhesive. For tests involving Scotch tape, the tape was cut into 40 mm × 19 mm strips and fixed on a flat surface using superglue, with the adhesive side facing upward.

For peel tests, VHB sheets were cut into long strips with a width ranging from 10 to 12.5 mm and a length of around 140 mm. Scotch tape has a width of 19 mm, and a length of around 140 mm is wrapped around either glass or steel tube. Two videos of peel tests, along with the corresponding load vs time curves in Figure S1a,b (Section I of the Supporting Information (SI)), respectively, show peeling of Scotch tape and VHB as Movies 1a and 1b at respective *v*_p_ = 10 and 0.2 mm/min. Adhesion between VHB or Scotch and the glass surface is established by bringing the adhesive into contact with the substrate under various conditions. For tensile adhesion and peeling tests presented in Figures 3a–c and 6, VHB was in contact with the glass tube for 5 min at 90 °C before testing. For the data in Figure 4, the contact time for both the tensile adhesion test and the poker chip was 10 min at 20 °C. For the data in Figure 5, peeling data involved a contact time of 30 min at 90 °C, while poker chip tests had both VHB and Scotch tape in contact with the glass tube for 10 min at 20 °C. For the tensile adhesion data in Figure 7, both substrates were in contact with VHB for 30 min at 90 °C. The strip length *L*_0_ for tensile adhesion tests varies from 10 to 12.5 mm on the glass tube and 11.5 mm on the steel tube.

### 2.3 Relationship between Tensile Adhesion and Poker Chip Tests

For adhesives such as Scotch tape with an adhesive layer as thin as 0.02 mm, only poker chip tests like that shown in Figure 1b are feasible for an approximate estimate of σ_adh_. In literature, a poker chip configuration with a thin layer of adhesive in contact with a probe is often employed for tack tests. The complicated fibrillation or cavitation^25,28^ makes it challenging to extract useful and pertinent information. Poker chip tests^29^ are typically characterized in terms of the work to debond (adhesive thickness multiplying the area under the stress vs strain curve),^23,25^ which has the same dimension as Γ_0_. To clarify the information revealed by poker chip tests, it is instructive for us to compare them with tensile adhesion tests since the interpretation of the latter is more straightforward and better-defined when debonding leaves nothing on the substrate, i.e., when the interfacial failure is entirely adhesive.

In both tensile adhesion and poker chip tests, shown in Figure 1a,b, rheological properties of a viscoelastic adhesive merely influence how fast the force *F* increases with time until detachment at *F*_c_, whereas debonding of adhesive from the substrate solely depends on interfacial interactions. Stretch rate λ̇ or speed *V* can be varied to evaluate adhesion strength σ_adh_ on different time scales at debonding in tensile adhesion tests and the peak nominal stress in poker chip tests. When adhesive failure prevails, poker chip tests reveal similar information. According to the analysis provided in Appendix A, we can estimate interfacial strength σ_interf_ using such probe tests to measure adhesion strength as

2 $$\sigma_{\mathrm{adh}}=F_{c}/\mathrm{BW}\;(\mathrm{tensile adhesion}),\mathrm{or},F_{\max}/\pi a^{2}\;\;(\mathrm{poker chip})$$

As shown in the following section, peeling tests reveal the same information on σ_adh_.

It is conceivable and perhaps even plausible that chain dynamics at the interface between adhesive and substrate can influence the interfacial strength: rapid chain movements might enable readsorption to offset any force-induced desorption. If such an effect existed or was dominant, interfacial strength could show a complicated temperature dependence.

### 2.4 Relationship between Peel Strength and Interfacial Strength

For 90° peeling, shown in Figure 1c, we bring adhesives onto a freely spinning wheel as sketched in Figure 2a, along with a photo of the actual apparatus in Figure 2b. For the present analysis, we take the fluctuating *v*_f_ to be around the mean value given by the probe (crosshead) speed *v*_p_. Peeling produces a force *F*_p_ acting on an area given by *WP*, where *P* can be comparable to adhesive thickness *B* in the presence of strong interfacial interactions—vanishing interfacial interactions are expected to result in diminishing *P*, decreasing all of the way to a monolayer scale, independent of *B*. The normalized force, often known as peel strength Γ_p_,

3 $$\Gamma_{p}=F_{p}/W$$

depends on σ_interf_ and *P*, as shown in eq 16 in Appendix B. Consequently, we further normalize Γ_p_ to introduce a potentially material-intrinsic quantity,

4 $$\sigma_{\mathrm{peel}}=F_{p}/\mathrm{WP}=\Gamma_{p}/P$$

and show it to be proportional to σ_interf_ in eq 17. We call σ_peel_ peeling stress.

In Section 3, we will present experimental results involving pressure-sensitive adhesives (VHB and Scotch tape) for probe/tack and peeling tests to explore the framework for polymer adhesion. At the foundation, we assert that Γ_p_ reflects the interfacial strength. In other words, as shown in Appendix B, σ_peel_ of eq 4 from peeling tests and σ_adh_ of eq 2 from tensile adhesion tests have the same origin, i.e., both related to σ_interf_. As shown in Figure 1c, peeling tests involve a characteristic length *P* whose magnitude largely depends on the strength of interfacial interactions and can be larger than adhesive thickness *B*. Thus, Γ_p_/*P* , not Γ_p_, may be more intrinsic to the pair of adhesive and substrate. The new dimensional analysis reveals (Γ_p_/*P*) ∝ σ_interf_ in place of Γ_p_ ∝ Γ_0_ in eq 1. Experiments in the following Section 3 support the basic concept that σ_peel_ defined by eq 4 is another measure of interfacial strength (cf. Appendix B). In other words, the peel strength of eq 3 can be expressed as

5 $$\Gamma_{p}∼\sigma_{\mathrm{adh}}P$$

Equation 5 indicates that any peeling speed *v*_p_ and temperature dependencies of Γ_p_ stem from those of adhesion strength σ_adh_, where *P* may be a weak function of speed and temperature. This conclusion differs from the prevailing view in the field of polymer adhesion, summarized in eq 1. When Γ_p_ involves a characteristic length scale *P* that is much greater than a monolayer scale, it far exceeds the magnitude of Γ_0_.

An instructive analogy exists between elastomeric fracture and adhesion, specifically, between tearing and peeling, both involving crack propagation and both operationally characterized in terms of a normalized force that shares the dimensions with the energy release rate or surface energy. In both cases, the force is higher at a higher speed and lower temperature. In both tearing and peeling, time and temperature effects arise, e.g., higher stress emergent on shorter time scales, because the crack propagation is an activated process. Moreover, a characteristic length scale emerges, either describing a stress saturation zone in bulk fracture^30^ or a region of size *P* at the peel front in interfacial fracture.

### 2.5 Interfacial Strength vs Substrate Surface Energy

Since Γ_p_ as a dimensionalized force has the dimension of work of adhesion, eq 1 is widely used to describe speed and temperature effects in peeling tests.^10^ There also exists an impression from the literature that substrates with higher surface energy γ_s_ should produce higher Γ_p_ because Γ_0_ varies with γ_s_. The phenomenological description based on eq 1 warrants several comments. First, the dimensional argument leading to eq 1 is likely inspired by the energy perspective in fracture mechanics, taking Γ_p_ to represent the energy released per unit area during peeling. But this proposal of Γ_p_ ∼ Γ_0_ lacks first principle-based reasoning. Second, Γ_0_ is much smaller than Γ_p_ and does not capture the fact that peeling involves stretching of chain network in the adhesive. In other words, peeling involves a destructive, non-equilibrium activation process. Third, peeling is a phenomenon of interfacial strength σ_interf_ being overcome by emergent stress. Thus, Γ_p_ reflects mechanical resistance in the form of eq 3 (force per length) or eq 4 (or force per area). Fourth, while using organic substrates assures that Γ_0_ remains at the level of γ_a_ ≈ γ_s_ on the order of tens of mJ/m^2^, even a clean (unoxidized) metallic substrate with γ_s_ ≈ 1 J/m^2^ does not usually result in Γ_0_ higher than ca. 0.1 J/m^2^. Since the relationship between γ_s_ and σ_interf_ is unclear, we can conduct tensile adhesion tests on different substrates (cf. Section 3.3) to shed some light.

### 2.6 Interfacial Debonding in Peeling and Probe/Tack Tests as an Activated Process

When adhesive–substrate interfacial interactions are established by physical adsorption alone, adhesion with higher cohesive strength involves only interfacial debonding. Adhesive failure is also an activated process because the interfacial detachment involves collective desorption or dissociation of noncovalent van der Waals bonds at multiple adsorption sites. Since debonding adhesives from substrate is an activated process, interfacial strength characterized by the degree and intensity of segmental adsorption on the substrate is both time- and temperature-dependent. We introduce a concept of lifetime *t*_intfS_ for interfacial interactions (adhesion) that is dependent on the state of adsorption. In the absence of any explicit discussion of the molecular picture, we assert that a polymer–substrate interface has finite lifetimes, and adhesion has finite strength as detachment amounts to complete removal of polymer–substrate contacts. It is stress, not the energy release rate, that dictates the lifetime. In the case of adhesion, the bonds are van der Waals in origin, and in the case of elastomeric fracture, carbon–carbon covalent bonds are typically involved.

To the leading order, the dependence of *t*_intfS_ on interfacial stress and temperature may be of the Arrhenius form,[Bibr newref32]

6 $$t_{\mathrm{intfS}}=t_{\mathrm{intfS}0}\exp\left[\frac{E_{\mathrm{intfS}}\left(\sigma_{\mathrm{adh}}\right)}{k_{B}T}\right]$$

where the energy barrier height *E*_intfS_ can be lowered during peeling and probe/tack tests. Inverting this equation, approximating *E*_intfS_ as *E*_0_ – *v*σ_adh_, like the treatment of solid fracture, [Bibr newref33],[Bibr newref34] we may find σ_adh_ to change logarithmically or very weakly with the characteristic time involved in the measurement of σ_adh_. Moreover, on the same time scale, eq 8 predicts that adhesion strength σ_adh_ and therefore peel strength Γ_p_ per eq 5 are both larger at lower temperatures.

Like our theory for elastomeric fracture,^30,31^ we theorize here that adhesive failure through interfacial debonding occurs when *t*_intfS_ decreases due to increasing interfacial stress until it meets the experimental time scale, characterized by the elapsed time at debonding, which is dictated by the applied rate, as shown below in eq 9.

In treating rubber friction against smooth solid surfaces, Schallamach[Bibr newref36] advanced the concept of rate-dependent detachment of polymer chains using reaction-rate theory of Eyring.[Bibr newref37] Peeling strength was suggested[Bibr newref38] to be a function both of the energy of interfacial bonds and of bulk energy loss in a viscoelastic adhesive. When an interfacial fracture involves chemical bonding of adhesives to a substrate, breaking of covalent bonds is involved. Chaudhury has treated such a case^32,33^ within the framework of Kauzmann–Eyring–Bell [Bibr newref32],^34^ to explain rate and temperature dependencies. To our knowledge, peeling speed and temperature effects on peeling strength for adhesion with physical contacts have not yet been interpreted in terms of an activated debonding process. On the other hand, Bell was among the first to treat cell adhesion as activated processes.[Bibr newref32]

## 3 Results and Discussion

Given the current consensus^20,25^ regarding the nature of polymer adhesion, e.g., characteristic speed and temperature dependencies of peel strength (cf. eq 1), we propose focusing on the essential case where peeling involves only adhesive failure. In the absence of cohesive failure, we show that tensile adhesion and poker chip tests, sketched in Figure 1a,b, disclose similar, equivalent information about interfacial strength. Therefore, we will first present in Section 3.1 results from tensile adhesion tests and compare them with those from conventional poker chip tests to confirm the meaning of poker chip tests and illustrate the origin of peel strength.

### 3.1 Probe/Tack Tests

#### 3.1.1 Tensile Adhesion Test

Availability of VHB sheets of finite thickness makes it possible to carry out a new test that we call “tensile adhesion”, as shown in Figure 1a. Movie 2 shows such a test at *V* = 200 mm/min with *L*_0_ = 14 mm, with its stress vs time curve in Figure S2 in Section II of the SI, indicating (a) significant partial interfacial detachment along the edge of width (*W* = 12.5 mm) occurs 33 s after the startup and complete detachment ensues 3 s later, (b) during this period of 3 s, engineering stress changes from 0.43 to 0.52 MPa. Visual inspection confirms that the tensile adhesion test involves only interfacial debonding. Given the time- and temperature-dependent nature of σ_adh_ described in Section 2.3, we perform tensile adhesion tests at various temperatures using different speeds so that debonding occurs on different time scales given by

7 $$t_{\mathrm{db}}=\left(\lambda_{\mathrm{db}}-1\right)/\mathrm{\lambda ̇},,\mathrm{with}\;\mathrm{\lambda ̇}=V/L_{0}$$

where λ_db_ is the stretch ratio of the adhesive strip at debonding and only weakly increases with λ̇. Therefore, we can control *t*_db_ (∼1/λ̇) by choice of *V* and *L*_0_. Such tests establish how interfacial interactions between the adhesive and substrate depend on time and temperature. Conversely, regarding *t*_db_ as a measure of *t*_intfS_ of eq 8, we explore how the lifetime of interfacial interactions depends on the applied interfacial stress.

For example, Figure 3a summarizes the raw data (tensile stress vs time curves), presented in Figure S3a–d in Section III of the SI, in terms of stress σ_adh_ according to eq 2 versus the time *t*_db_ at debonding per eq 6 . It is clear from Figure 3a that σ_adh_ is systematically lower on longer time scales and increases with decreasing temperature. Like the phenomenon of elastic rupture,^31^ which also involves an activated process^30^ associated with covalent bond dissociation, there is time–temperature equivalence (TTE), where the lifetime *t*_intfS_ of polymer–substrate interactions is the hidden internal clock. It is conceivable that polymer dynamics can influence lifetime *t*_intfS_ if the kinetics of polymer adsorption and stress-induced desorption can affect the barrier height *E*_0_ in eq 8.

Since adhesive failure takes place when *t*_intfS_ = *t*_db_, tensile adhesion tests provide a measure of *t*_intfS_ as a function of applied stress. While the concept of TTE per eq 8 is well-defined for a fixed interfacial stress, TTE cannot be used to perform a horizontal shift along the time axis in Figure 3a—each of the four sets of σ_adh_ vs *t*_db_ involves a range of interfacial stress. On the other hand, we can forcefully shift the time scale for the four sets of data in Figure 3a to form an approximate master curve in Figure 3b. The shift factor *A*_T_ (circles), loosely representing *t*_intfS_(*T*)/*t*_intfS_(*T*_ref_), in Figure 3c still encompasses some profound information. It approximately reveals how the lifetime *t*_intfS_ increases with decreasing temperature. Since higher interfacial stress (σ_adh_) is involved at lower temperatures, we can expect the temperature dependence of *t*_intfS_ to be stronger than that shown in Figure 3c, i.e., changing more than three decades from 22 to 80 °C. Also plotted in Figure 3c are data points from a second shift factor *A*_T(peel)_ in squares that is used to shift σ_peel_ data along the time axis for peeling tests, as presented below in Section 3.2.

Taking *t*_db_ of eq 8 as representing the lifetime *t*_intfS_, the concept of TTE allows us to explore the dependence of *t*_intfS_ on interfacial stress σ_adh_ over a much wider range, as shown in Figure 3b. Approximately, *t*_db_ (regarded as *t*_intfS_) shows an exponential dependence on σ_adh_ in agreement with eq 8, where the stress dependence of the energy barrier *E*_intf_ may be linearized as *E*_intf_ = *E*_0_ – *v*σ_adh_.

**Figure 3:**
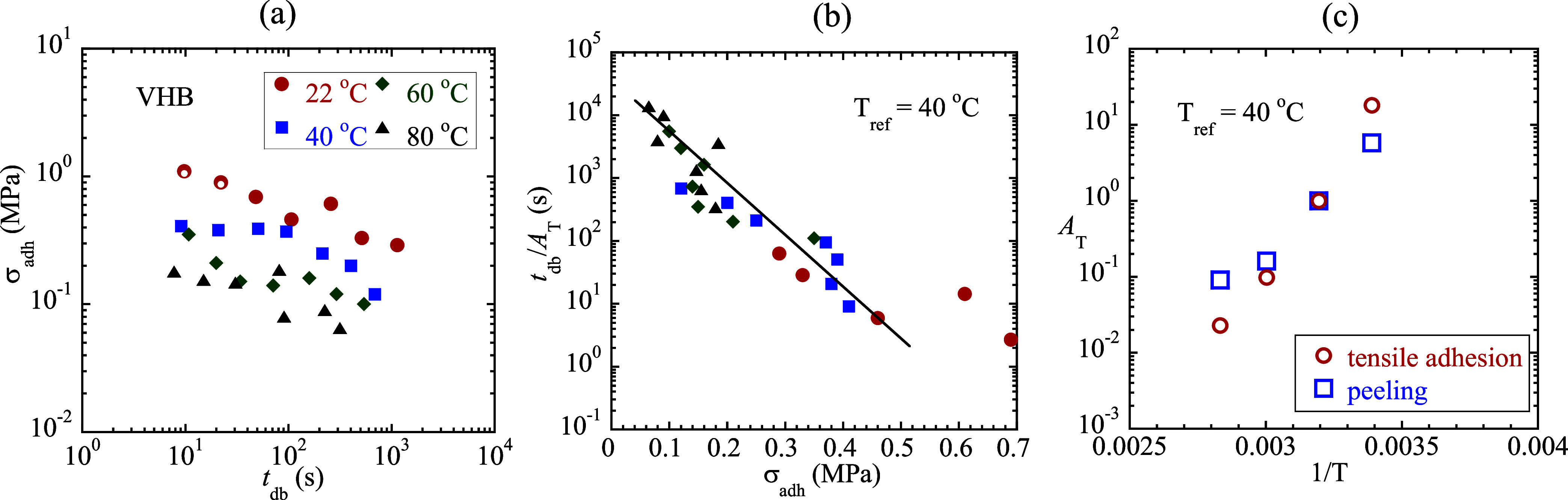
(a) Engineering stress at debonding from tensile adhesion tests, read from Figure S3a–d in the SI, where the two open circles indicate cohesive failure. (b) “Master curve”, constructed from (a) by shifting along the time axis. (c) Shift factors *A*_T_ as a function of temperature, used in the construction of (b) and in Figure 6.

#### 3.1.2 Poker Chip Test

Results from tensile adhesion tests allow us to clarify the meaning of poker chip tests. Figure 4a shows that both poker chip (open symbols) and tensile adhesion tests (filled symbols) produce similar adhesive failure, although tensile adhesion produces a cleaner interpretation of the nature of interfacial detachment. The comparison largely shows the same trend: On longer time scales, σ_adh_ of eq 2, is lower. In poker chip tests, debonding first takes place at the weakest site/region of adsorption. Approximately, the peak stress signals the onset of detachment. The agreement between tensile adhesion and poker chip tests is shown in filled and open circles in Figure 4b, indicating that the poker chip test is valid as an approximate setup to characterize adhesion.

**Figure 4:**
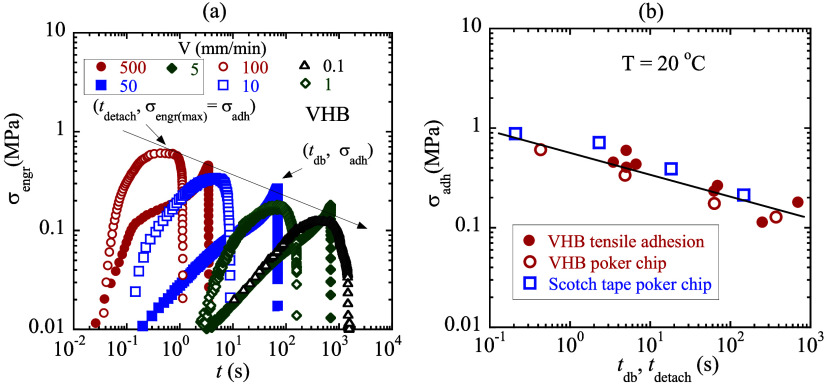
(a) Engineering stress vs time from both tensile adhesion tests (boxed in three filled symbols) and poker chip tests (four open symbols), involving crosshead speed *V* from 500 to 0.1 mm/min, where elapsed times at stress peak are denoted by either *t*_detch_ or *t*_db_. In tensile adhesion tests (filled symbols), specimen length *L*_0_ = 10.5, 10.6, and 5 mm, respectively, for *V* = 5, 50, and 500 mm/min. The arrow shows the trend: σ_adh_ decreases with decreasing *V*. (b) Adhesion strength σ_adh_, read from the peak stresses in (a) at either *t*_detach_ (open circles) in poker chip tests or at *t*_db_ (filled circles) for debonding in tensile adhesion tests. Also plotted is σ_adh_ from poker chip tests on Scotch tape (open squares) for comparison with open circles (VHB).

Upon such validation of poker chip tests, we can demonstrate the essence of peeling tests in Section 3.2 by comparing a thick adhesive, such as VHB, with a thin one, such as Scotch tape that is only amenable to characterization by poker chip tests. The present Scotch tape happens to show interfacial strength comparable to that of VHB when bare glass is the common substrate. Specifically, reading from the poker chip tests on Scotch tape, presented in Figure S4a,b in Section IV of the SI, open squares in Figure 4b show only slightly higher magnitudes than open circles of VHB.

### 3.2 Peeling Tests

Peeling behavior of adhesives is described by both the emergent force, normalized as Γ_p_ per eq 2, and a characteristic length *P* shown in Figure 1c. According to our analysis in Section 2.4, Γ_p_ is not a material function because it varies with adhesive thickness *B*. Using the two adhesives with remarkably different thicknesses in VHB and Scotch tapes but similar interfacial strength, we make a comparison in Figure 5a between them based on the raw data, i.e., peel strength vs time in Figure S5a,b in Section V of the SI. At each speed, we measure in “steady state” the characteristic length *P* and peel strength Γ_p_, as shown in Figure 5a, based on video recording of peeling, e.g., images in Figure S6a,b in Section VI of the SI, whereas Figure S6c,d compares the front geometries at two speeds. Here, higher Γ_p_ at higher *V* occurs without greater stretching of VHB because VHB shows^35^ stronger stress response at higher rates. Although Figure 4b shows comparable adhesion strength σ_adh_, Γ_p_ of these two adhesives are markedly different. Notably, the much higher Γ_p_ of VHB also has proportionally higher *P* due to higher thicknes.

The much higher Γ_p_ of VHB stems from the fact that adhesion occurs over a much larger area of *WP*, with *P* of VHB being ten times *P* of Scotch tape, as shown in Figure 5a. The similar interfacial strength of VHB and Scotch tape on the glass surface should reveal similar intrinsic characteristics. The peeling stress σ_peel_ of eq 4 instead of the Γ_p_ of eq 2 is the quantity to describe interfacial interactions in peeling tests. Figure 5b shows that both VHB and Scotch tape show comparable peeling stress (filled symbols) and adhesion strength (open symbols) from poker chip tests over a wide range of comparable time scales, illustrating the origin of peel strength, i.e., validating eq 5. The difference in adhesion conditions for peeling and poker chip tests evidently did not result in any significant difference in interfacial strength. Figure 5b also verified that the magnitudes of σ_peel_ and σ_adh_ for VHB are comparable to those of Scotch tape. Here for the comparison between σ_peel_ and σ_adh_, at peeling speed *v*_p_, taken to equal the crosshead speed *V*, a pertinent time scale is identified as *P*/*v*_p_, so that the two stresses can be evaluated on the same footing in Figure 5b. Since these time scales originate from bond lifetime *t*_intfS_ of eq 8, we replot Figure 5b in Figure 5c, showing indeed that they undergo exponential decrease as a function of stress. The data should not be extrapolated to zero stress on this semilogarithmic plot since a stress threshold is expected: Detachment on any realistic time scale would require nonvanishing stress.

Peeling may be regarded as fracture-mechanical, requiring a characteristic stress and length to describe, as shown in eq 5. Consequently, peeling of the two different adhesives (VHB and Scotch tape) is described by the peel strength, given by the product of comparable adhesion strength but very different length *P*. The approximately 1 order-of-magnitude difference in Γ_p_ in Figure 5a stems from the corresponding difference in *P*.

**Figure 5:**
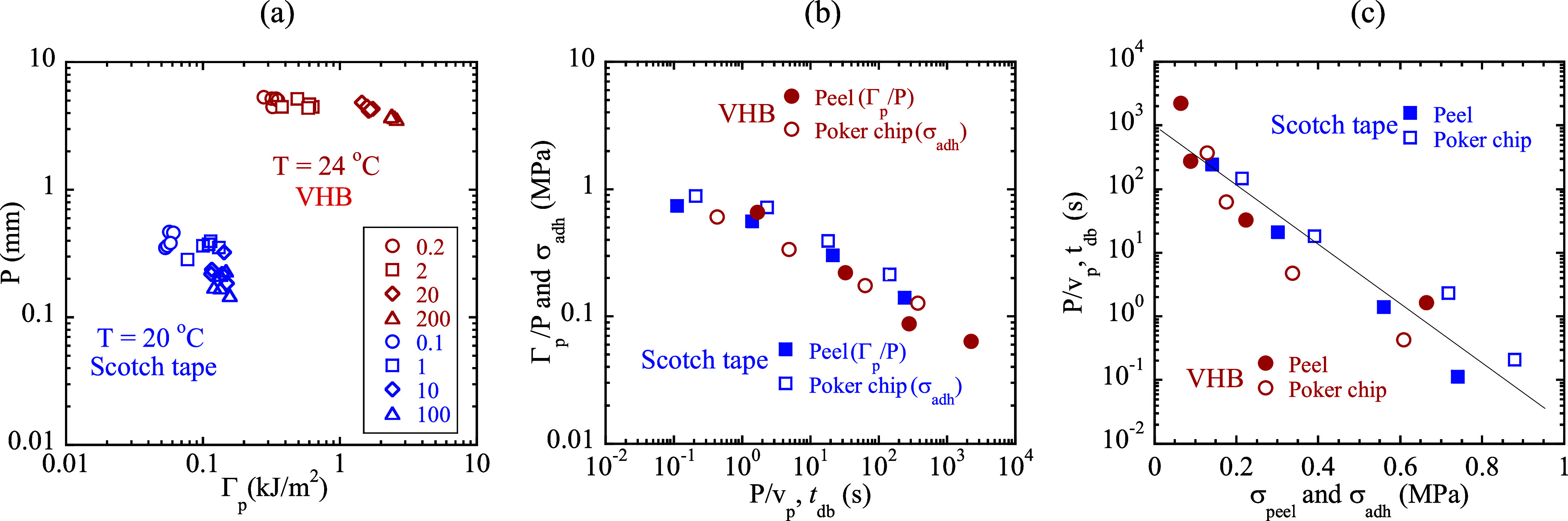
(a) Characteristic length *P* vs peel strength Γ_p_ defined by eq 3. (b) Plotting the data in (a) in terms of the intrinsic quantity–peeling stress per eq 4, along with adhesion strength from Figure 4b, as a function of the characteristic time, given by either *P*/*v*_p_ or debonding time *t*_db_. (c) Characteristic times as a function of adhesion and peel strengths.

To examine the temperature effect on σ_peel_, we carry out 16 peeling tests at four temperatures with four rates at each temperature. Figure 6 summarizes the raw data and preliminary analysis presented in Figures S7a–d, S8a–d and S9 in the SI (respectively, in Sections VII, VIII, and IX of the SI). Like Figure 3b, the master curve in open symbols in Figure 6 involves a horizontal shift of data in Figure S9 involving a shift factor *A*_T(peel)_, i.e., squares in Figure 3c. Also plotted in Figure 6 are the same data points from Figure 3b, in filled symbols. Since the two straight lines are drawn to have the same slope, Figure 6 shows σ_peel_(*P*/*v*_p_ = *t*_db_) = σ_adh_(*t*_db_), thus revealing the meaning of peeling tests and demonstrating that time and temperature dependencies of peel strength stem from those of interfacial interactions, not from the bulk viscoelasticity of adhesives: Since tensile adhesion tests involve only adhesive failure yet reveal the same dependencies, we conclude that the same physics is at play, i.e., the speed and temperature effects in peeling tests stem from the dependence of interfacial strength on temperature that also varies on different time scales.

**Figure 6:**
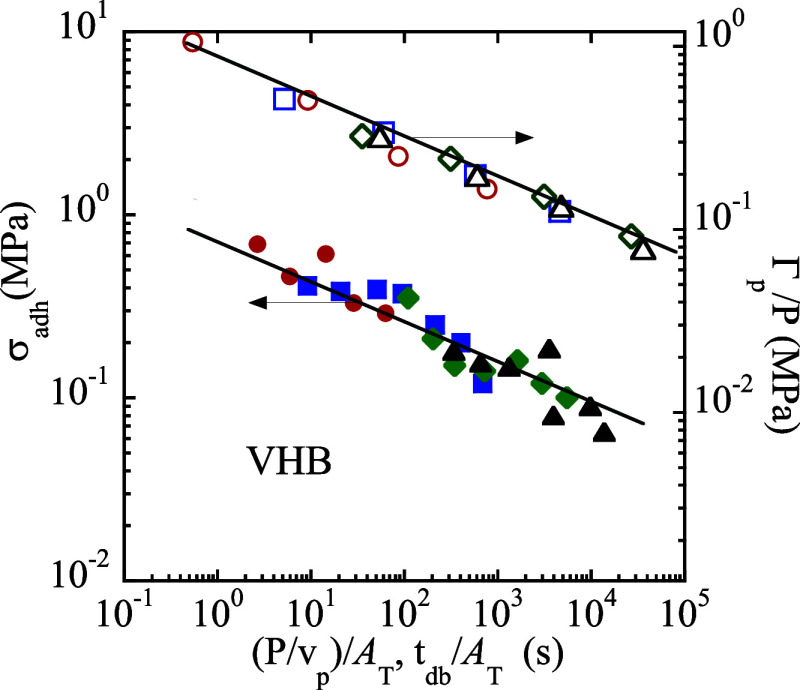
Comparison between the master curve of peeling stress σ_peel_ (right-hand-side *Y-*axis from 1.2 × 10^–3^ to 1.2) against (*P*/*v*_p_)*A*_T(peel)_ and that of adhesion strength σ_adh_ (left-hand-side *Y*-axis from 1.2× 10^–2^ to 12) against *t*_db_ *A*_T_ at different temperatures, where *A*_T(peel)_ and *A*_T_ are, respectively, the squares and circles in Figure 3c. Double *Y*-axes are employed to displace one master curve from the other by one decade to avoid overlap.

The agreement between σ_adh_ and Γ_p_/*P* in Figures 5c and 6 for either VHB or Scotch tape configurations can be understood by comparing eqs 13 and 17, both proportional to interfacial strength with prefactors on the order of unity.

### 3.3 Surface Energy of Substrate vs Adhesion Strength

Section 2.5 indicates that interfacial strength has no direct relationship with the substrate’s surface energy. Although the surface energies of Mylar as well as Kapton, glass, and steel may differ significantly, from 30 to 1000 mJ/m^2^, there is no theory to describe how their interfacial interactions with VHB vary. Figure 7, read from Figure S10 in Section X of the SI, shows that the adhesion strengths are within a factor of 3 over two decades of time scale. Similarly, peel strength is only lower on the steel tube than on the glass tube at room temperature, as shown in Figure S11 in Section XI of the SI. Since appreciable pressure is applied to establish contact between VHB and the steel tube, we have assumed that VHB achieved comparable access to the steel surface due to adequate pressure. Thus, the surface roughness of the steel tube may not be the reason for the observed lower adhesion strength—the oxidative layer on the steel tube may be the origin. In contrast, Mylar and Kapton films should be very smooth down to submicrons. Thus, the results in Figure 7 indicate a certain correlation between interfacial strength and surface energy of the substrate. Further exploration of the relationship between the surface energy of substrate and adhesion strength is an interesting topic for future study, but beyond the scope of the current work. Because of the correlation between σ_adh_ and Γ_p_, demonstrated in Figure 6and eq 5, it is reasonable to conclude that peel strength follows the same trend as described by Figure 7. In fact, since *P* decreases with adhesion strength, peeling strength on these plastic surfaces can be expected to be significantly lower than indicated by these tension adhesiont tests.

**Figure 7:**
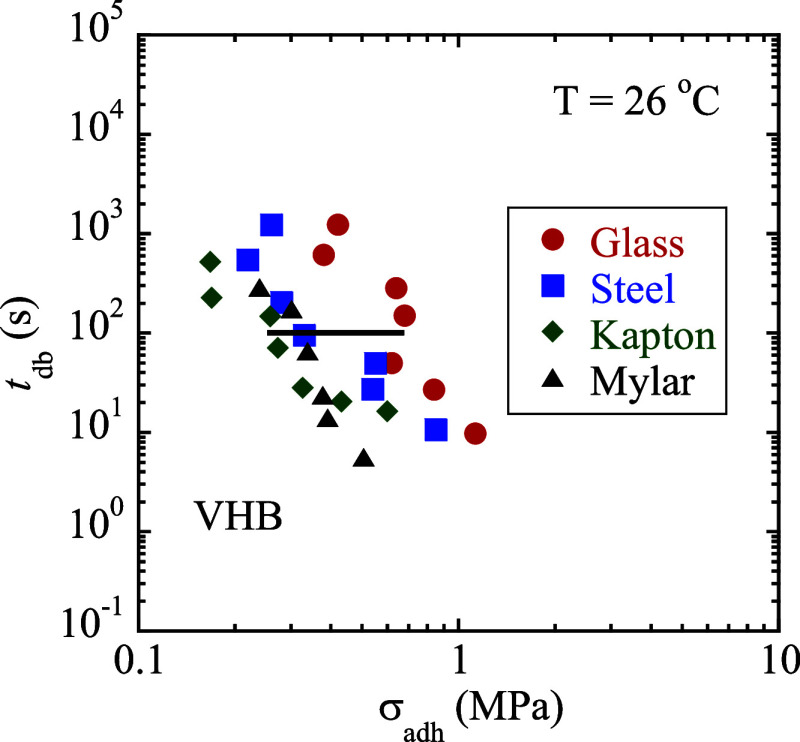
Debonding time from tensile adhesion tests at room temperature (26 °C) as a function of adhesion strength σ_adh_ on four different substrates, made of glass, steel, Kapton (PI), and Mylar (PET), respectively. The horizonal line shows the spread in σ_adh_ of ca. 3 on a given time scale.

## 4 Conclusions

Departing from the conventional description of polymer adhesion, we show that time and temperature effects in peeling tests can be understood in terms of interfacial strength and a characteristic length scale that defines the area of active stress at the peeling front. Like adhesive failure in tensile adhesion tests (cf. Figure 1a), peeling (cf. Figures 1c and 2a,b) amounts to stress-induced interfacial debonding of van der Waals bonds between adhesive and solid substrate. Since debonding is an activated interfacial process, both probe and peeling tests show similar time and temperature dependencies. Specifically, higher peel strength at higher peeling speeds and lower temperatures stems from the fact that only higher interfacial stress can cause interfacial debonding to take place on shorter time scales and at lower temperatures. In other words, the time- and temperature-dependent phenomenology of peel strength Γ_p_ has a different origin from that proposed based on energy dissipation associated with viscoelastic bulk deformation of adhesives. In the literature, the influence of interfacial interactions on Γ_p_ has been mentioned^41^ within the phenomenological framework of eq 1. But the present viewpoint contrasts with the prevailing interpretation of the nature of polymer adhesion, which regards peel strength Γ_p_ in eq 3 as energy per unit area.

Making a connection between tensile adhesion and peeling tests has permitted us to conclude that Γ_p_ is merely a mechanical quantity, measuring the magnitude of a partially normalized force associated with interfacial interactions on a given time scale and at a temperature of choice. Probe/tack tests (e.g., tensile adhesion tests) and peeling tests describe the same physics, characterized by interfacial strength.

Explicitly, our experiments show that interfacial failure in tensile adhesion tests is the same process of detachment of adhesive from solid substrate as that involved in peeling. In both cases, the strength varies with the temperature on different time scales. Since the interfacial detachment is adhesive rather than cohesive, it is straightforward to conclude that any observed rate and temperature dependencies reflect interfacial bonding as an activated process.[Bibr newref32] Viscoelastic properties of adhesives in such a test merely dictate how quickly tensile stress builds up and generates interfacial stress all of the way up until interfacial strength is reached. It is beyond the scope of the current study to explore any relationship between the network structure^36^ of adhesives and their interfacial strength with solid substrates.

To reiterate, when peeling involves only adhesive failure, the same physics is involved in peeling as in the tensile adhesion test. Specifically, both normalized peel strength, i.e., peeling stress, and adhesion strength, are comparable on the same time scales at various temperatures. The strength of interfacial interactions between adhesive and solid substrates varies with the surface energy of the substrate, based on our measurement of adhesion strength on four different substrates. The generality of this conclusion remains to be further explored. Since interfacial interactions are different for different substrates, it is already known^37^ that surface treatment can produce considerable changes in Γ_p_. We plan to describe the effect of surface treatment in our next paper.

Like our treatment of polymer fracture,^30^ where we take the stress perspective and recognize how local stress and characteristic length combine to describe fracture, we have shown here that polymer adhesion in the case of peeling can be similarly understood in terms of interfacial strength σ_interf_ and pertinent length scale *P*, in contrast to the conventional description based on work of adhesion, which is a fracture mechanics-motivated, energy-based perspective. Here *P*, crudely related to Γ_p_ according to eq 5, is not the elasto-adhesive length^25^,[Bibr newref46] defined by Γ_p_/*E*. In both elastomeric fracture and adhesion, the energy perspective can only interpret the rate and temperature effects in terms of viscous energy dissipation when materials only undergo predominantly elastic deformation before fracture and detachment. Our stress perspective naturally encompasses cases where Γ_p_ ≫ Γ_0_. We have not only explained why peel strength may vary with peeling speed and temperature but also indicated that interfacial strength dictates its magnitude along with a characteristic length *P*.

By explaining time and temperature effects on polymer adhesion in terms of the lifetime of interfacial interactions, this work revives previous concepts [Bibr newref32],[Bibr newref36],[Bibr newref38] of kinetics of interfacial interactions to elucidate the nature of polymer adhesion. The findings have direct implications for the design of adhesive interfaces, soft coatings, and polymer–solid contacts through physical adsorption, where interfacial debonding leads to adhesive failure. The present alternative framework explored may be equally relevant and plausibly applicable to the adhesion of all soft materials whose cohesive strength is higher than the adhesion strength.

The present conclusions also have implications regarding the adhesion theory based on the JKR^2,38^ model for a spherical body (of radius *R*) in contact with a flat substrate. When contact between a rubber sphere and a hard solid surface involves strong physical absorption, detachment may involve a magnitude of the pull-off force *F*_c_ much greater than *F*_JKR_ ∼ Γ_0_ *R*. In this case, comparison with peeling tests should produce insightful results. In the case of adhesive detachment, time and temperature effects on *F*_c_ are plausibly interfacial in origin, not related to Dupré work of adhesion and/or bulk viscoelastic properties. Such a viewpoint offers a complementary perspective to the standard energy perspective, in which Persson and co-workers have attempted^39^ to generalize the JKR approach by associating adhesion energy (peel strength) with viscous energy dissipation. In the case of peeling, we show in the present work that the origin of such time and temperature effects is activation of interfacial interactions, not viscoelastic dissipation. The separation of two elastic bodies in physical contact also amounts to overcoming interfacial strength, involving *F*_c_/*R* naturally much greater than Γ_0_. Thus, future studies of time and temperature effects^41,36,40^ on *F*_c_ (≫*F*_JKR_) may benefit from our stress perspective.

In summary, although our experiments utilize two common pressure-sensitive adhesives and four substrates, they reveal a framework that may be universal when the substrate is a smooth solid surface. Whenever peeling results in adhesive failure, the process is fundamentally interfacial; the increase in the peel strength observed at higher speeds or lower temperatures stems from the thermally activated dissociation of interfacial bonding. When the substrate is also made of a polymer network, as often studied,^36^ the nature of interfacial interactions can be more complicated. Time and temperature effects may arise from polymer dynamics at the interface, where entanglement due to chain uncrossability can influence interfacial strength.

## Supplementary Material









## Appendix A: Interfacial Strength Measured by Probe Tests

We show here that interfacial strength dictates adhesion strength regardless of whether stress intensification occurs at the edges in the tensile adhesion setup shown in Figure 1a. Since *W* ≫ *B* = 1 mm, we can carry out the following simple analysis to relate nominal stress to interfacial strength, which is overcome upon detachment. The total tensile force at detachment is related to critical load *K*_c_ through the following integration, acknowledging that the interfacial stress field varies as σ­(*x*) = *K*_c_/[(*B*/2–*x*) + ρ]^1/2^, with ρ representing a Creager–Paris^42^-like cutoff length,

A.1
$$F_{c}=2W\int_{0}^{B/2}\sigma \left(x\right)\;dx=4\mathrm{WB}\left(K_{c}/B^{1/2}\right)\left[{\left(1/2+\rho /B\right)}^{1/2}-{\left(\rho /B\right)}^{1/2}\right]$$

so that

A.2
$$\sigma_{\mathrm{adh}}=F_{c}/\mathrm{WB}=A\left(\rho /B\right)\sigma_{\mathrm{interf}}$$

where the prefactor *A* is a function of the geometry, i.e., the ratio α = ρ/*B*, given by

A.3
$$A\left(\alpha \right)=4\alpha^{1/2}\left[{\left(1/2+\alpha \right)}^{1/2}-\alpha^{1/2}\right]$$

In eq 10, critical stress intensity factor *K*_c_ is related to interfacial strength σ_interf_ as

A.4
$$K_{c}=\rho^{1/2}\sigma_{\mathrm{interf}}$$

This expression resembles the Inglis solution applied^30^ to describe a fracture from a crack of sharpness ρ when cohesive strength is exceeded at crack tip. Although ρ is unknown, we expect α to be on the order of unity because VHB is highly stretchable, so that σ_adh_ may be comparable to σ_interf_. With eq 11, the origin of *F*_c_ (or σ_adh_) is shown to be interfacial.

Poker chip tests can be similarly described. In other words, assuming stress divergence is described by σ­(*r*) = *K*_c_/(*a* – *r*)^1/2^, *F*_max_ is given by

A.5
$$F_{\max}=\int_{0}^{a}\sigma \left(r\right)2\pi r\;dr=\left(8\pi /3\right)K_{c}a^{3/2}=\pi a^{2}\left(8K_{c}/3a^{1/2}\right)$$

so that

A.6
$$\sigma_{\mathrm{adh}\left(\mathrm{pc}\right)}=F_{\max}/\pi a^{2}=\sigma_{\mathrm{interf}}\left(8/3\right){\left(\rho /a\right)}^{1/2}$$

where *a* = 3 mm in the present study. Equation 15 is consistent with the argument^43^ based on contact mechanics and reveals that the critical stress is related to interfacial strength.

## Appendix B: Interfacial Origin of Peeling Strength

Similarly, peeling strength depends on interfacial strength σ_interf_. Peeling at an imposed speed *v*_p_ involves overcoming interfacial bonding on a time scale set by *v*_p_. The mechanical resistance to debonding is measured as the tensile force *F*_p_, related to the emergent interfacial stress σ­(*x*) = *K*_c_ *x* ^–1/2^ as

B.1
$$\Gamma_{p}=F_{p}/W=\int_{0}^{P}\sigma \left(x\right)\;dx=2K_{c}P^{1/2}$$

so that

B.2
$$\Gamma_{p}/P=\sigma_{\mathrm{peel}}=\sigma_{\mathrm{interf}}{\left(4\rho /P\right)}^{1/2}$$

Like expressions shown by eqs 11 and 15, eq 17 reveals the interfacial origin of peel strength. Here, we represent the characteristic Inglis length with the same symbol ρ for probe/tack and peeling tests. When ρ is in the range of a millimeter, adhesion strength and peeling stress are comparable, given the actual values of *B*, *a*, and *P* in the present study. Moreover, it is plausible that ρ is proportional to *P*. Otherwise, we could consider *F*_p_/*W*(ρ*P*)^1/2^ as a more exact estimate of interfacial strength. Finally, in either the refined description given in eqs 16 and 17 or eqs 3 to 5, peel strength depends on interfacial strength.

In these two Appendices, we assume a certain form of integrable stress singularity at contact lines with the purpose of showing that the nominal detachment forces arising in tensile adhesion and peeling tests originate from overcoming interfacial strength. These forces explicitly depend on σ_interf_, with prefactors dependent on geometric parameters.

## Notes

Movie 1a: Real-time peeling process of Scotch tape corresponding to Figure SI.1a, recorded at a speed of *v*_p_ = 10 mm/min (MP4)
Movie 1b: Real-time peeling process of VHB corresponding to Figure SI.1b, recorded at a speed of *v*_p_ = 0.2 mm/min (the video is sped up by a factor of 100) (MP4)
Movie 2: Real-time tensile adhesion test of VHB corresponding to Figure SI.2, recorded at a speed of *v*_p_ = 200 mm/min with birefringence imaging, showing the stress evolution and onset of interfacial debonding (MP4)
Tensile adhesion, poker chip, and peel test results; stress–time curves; photographs of peeling fronts used to determine *P*; temperature-dependent adhesion measurements; and relationships between *P*, Γ_p_/*P*, and *P*/*v*_p_ on different substrates (PDF)

## List of Symbols

*A*_T_: shift factor used to make master curve in Figure 3b
*B*: thickness of the sample
*E*_intfS_: energy barrier height_,_ defined in eq 6
*F*: tensile force measured in probe and peeling tests
*L*_0_: initial length of the sample
*P*: characteristic length at peeling front shown in Figure 1c
T_ref_: reference temperature
*t*_db_: time at debonding from substrate in tensile adhesion tests, given in eq 7
*t*_detach_: time at tensile force maximum in poker chip tests
*t*_intfS_: lifetime of interfacial interactions (adhesion), introduced in eq 6
*V*: speed of stretching in tensile adhesion and poker chip tests
*v*_p_: peeling speed
*W*: width of the sample
Γ_0_: Dupré work of adhesion
Γ_p_: peel strength in eq 3
γ_a_: surface energy of adhesive
γ_s_: surface energy of substrate
γ_as_: adhesive–substrate interfacial energy
λ̇: stretch rate
λ_db_: stretch ratio of adhesive at debonding in tensile adhesion test
σ_adh_: adhesion strength, defined in eq 2
σ_interf_: interfacial strength
σ_peel_: peeling stress, defined in eq 4, as “normalized” peel strength
