# Supplementary material for: Adhesion Depends on Interfacial Strength: Time and Temperature Effects
Source: Langmuir. 2026 Jun 18;42(26):19108–17. doi: 10.1021/acs.langmuir.6c02054 (PMC13352635; doi:10.1021/acs.langmuir.6c02054)
Supplement: Supplementary file 1 [file la6c02054_si_004.pdf]

Supporting Information

Adhesion Depends on Interfacial Strength: time and temperature effects

Shi-Qing Wang*, Zehao Fan[1], Tianji Pang[1], and Zhe Cui

School of Polymer Science and Polymer Engineering

University of Akron, Akron, Ohio 44325

Number of pages: 8

Number of figures: 11

Number of schemes: 0

Number of tables: 0

* Corresponding author at swang@uakron.edu

Contents

I. Stress-time curves of typical Scotch tape and VHB peeling..S3

II. Stress-time curve for tensile adhesion test on VHB. S3

III. Tensile adhesion test on glass at various temperatures. S4

IV. Poker chip test of Scotch tape and VHB. S4

V. Peel test of Scotch tape and VHB...S5

VI. Photos of the peeling front to determine P S5

VII. Peel test of VHB at different temperatures. S6

VIII. Relationship between P and Γ/P from peel test of VHB at different temperatures. S7

IX. Relationship between Γ/P and **P/vp** from peel test of VHB at different temperatures.....S7

X. Tensile adhesion test of glass and metal...S8

XI. Relationship between Γ/P and **P/vp** from peel test of VHB on different substrates at different temperatures..S8

I. Stress-time curves of typical Scotch tape and VHB peeling.

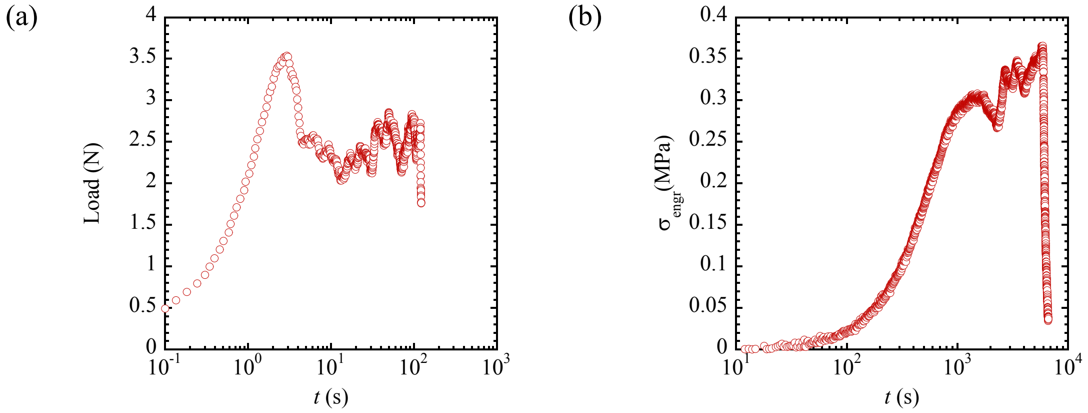

Figure SI.1 Stress-time curves of typical (a) Scotch tape and (b) VHB peeling, corresponding to respectively Movie 1a with v_p = 10 and Movie 1b with v_p = 0.2 mm/min, which is sped up 100 times. Movie 1a appears faster because of greater magnification.

Movie 1a: Real-time peeling process of Scotch tape corresponding to Figure SI.1a, recorded at a speed of v_p =10 mm/min.

Movie 1b: Real-time peeling process of VHB corresponding to Figure SI.1b, recorded at a speed of v_p = 0.2 mm/min. The video is sped up by a factor of 100.

II. Stress-time curve for tensile adhesion test on VHB.

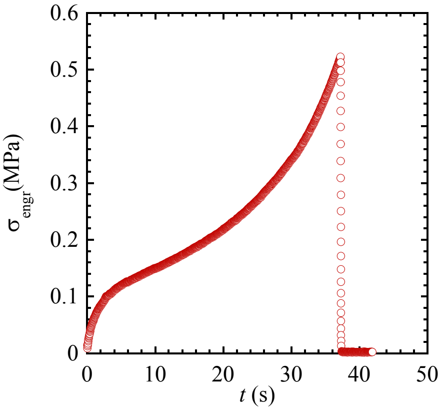

Figure SI.2 Stress-time curve for tensile adhesion test on VHB, corresponding to Movie 2 with v_p = 200 mm/min with birefringence, with W = 12.5 mm and L_0 = 14 mm.

Movie 2: Real-time tensile adhesion test of VHB corresponding to Figure SI.2, recorded at a speed of v_p = 200 mm/min with birefringence imaging, showing the stress evolution and onset of interfacial debonding.

III. Tensile adhesion test on glass at various temperatures.

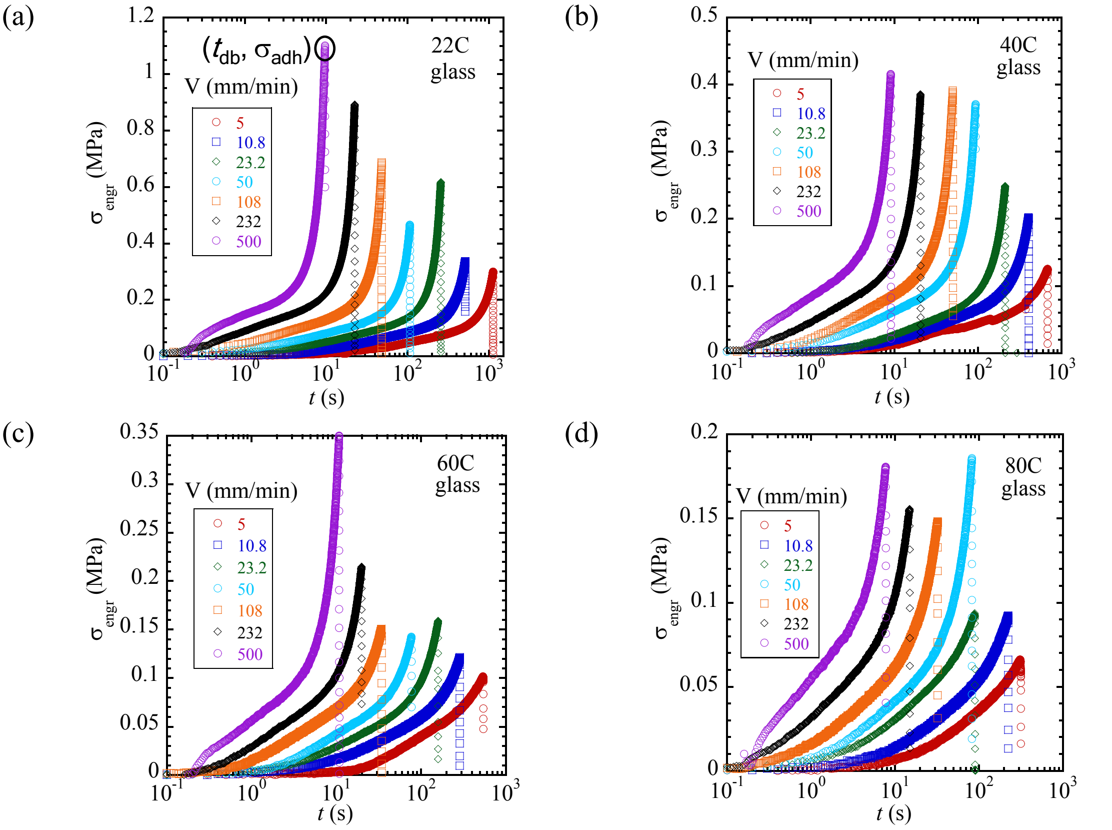

Figure SI.3a-d Tensile adhesion test on glass at various temperatures. Samples were adhered to glass at 90C for 5mins prior to experiment.

IV. Poker chip test of Scotch tape and VHB.

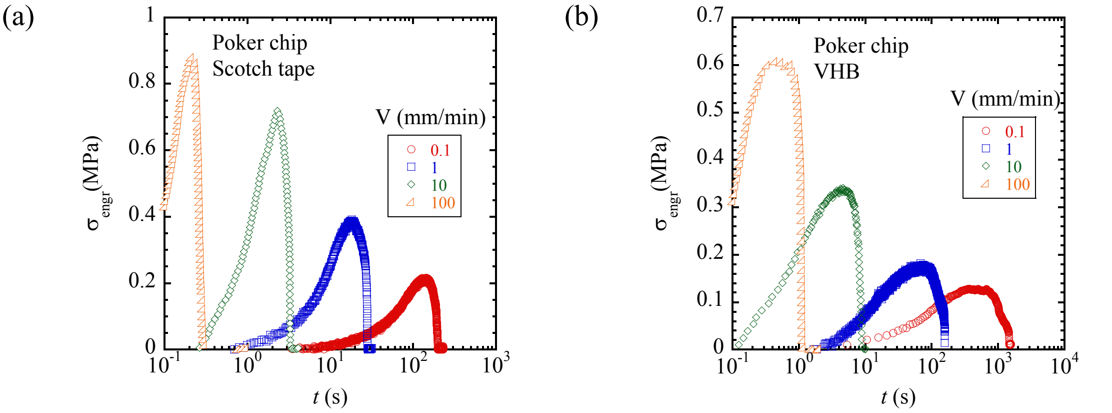

Fig. SI.4a-b Poker chip of Scotch tape and VHB. Samples were adhered to glass at 90C for 30mins prior to experiment.

V. Peel test of Scotch tape and VHB.

(a)

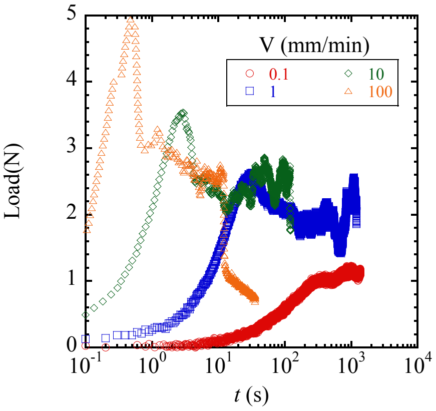

(b)

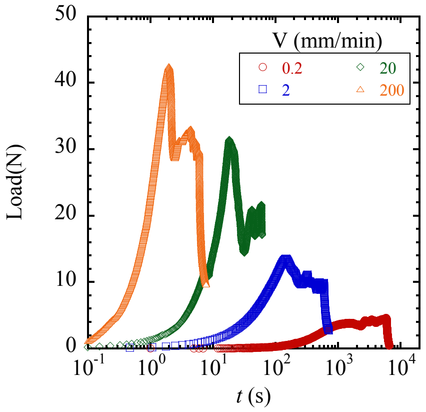

Fig. SI.5 Peel test of (a)Scotch tape and (b)VHB, plotted in load-time mode. Samples were adhered to glass at 90C for 30mins prior to experiment.

VI. Photos of the peeling front to determine P

(a)

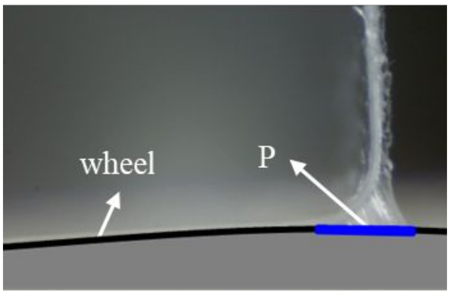

(b)

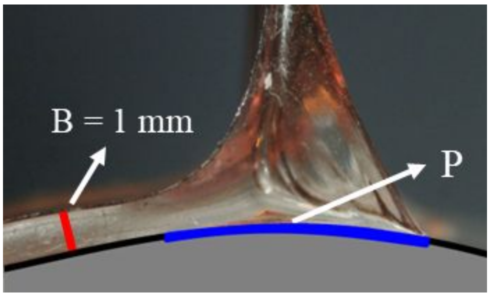

V = 200 mm/min (F =30 N)

(c)

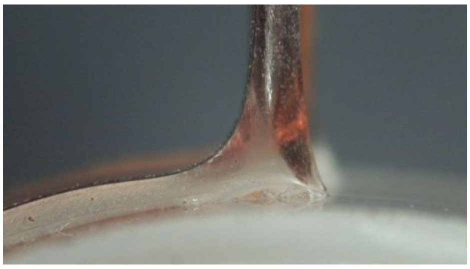

V = 0.2 mm/min (4.4 N)

(d)

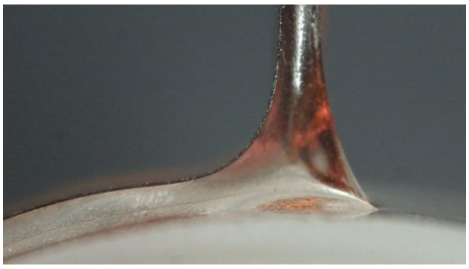

Figure SI.6a-b Illustration of P during peeling. P is defined as the blue curve of the peeled strip at the peeling front. (a) Side view of the peeling configuration of Scotch tape on the wheel. (b) Side view of the peeling configuration of VHB on the wheel. Peeling of VHB at speeds (c) 200 and (d) 0.2 mm/min, which are also the peeling speed v_p in steady state.

VII. Peel test of VHB at different temperatures.

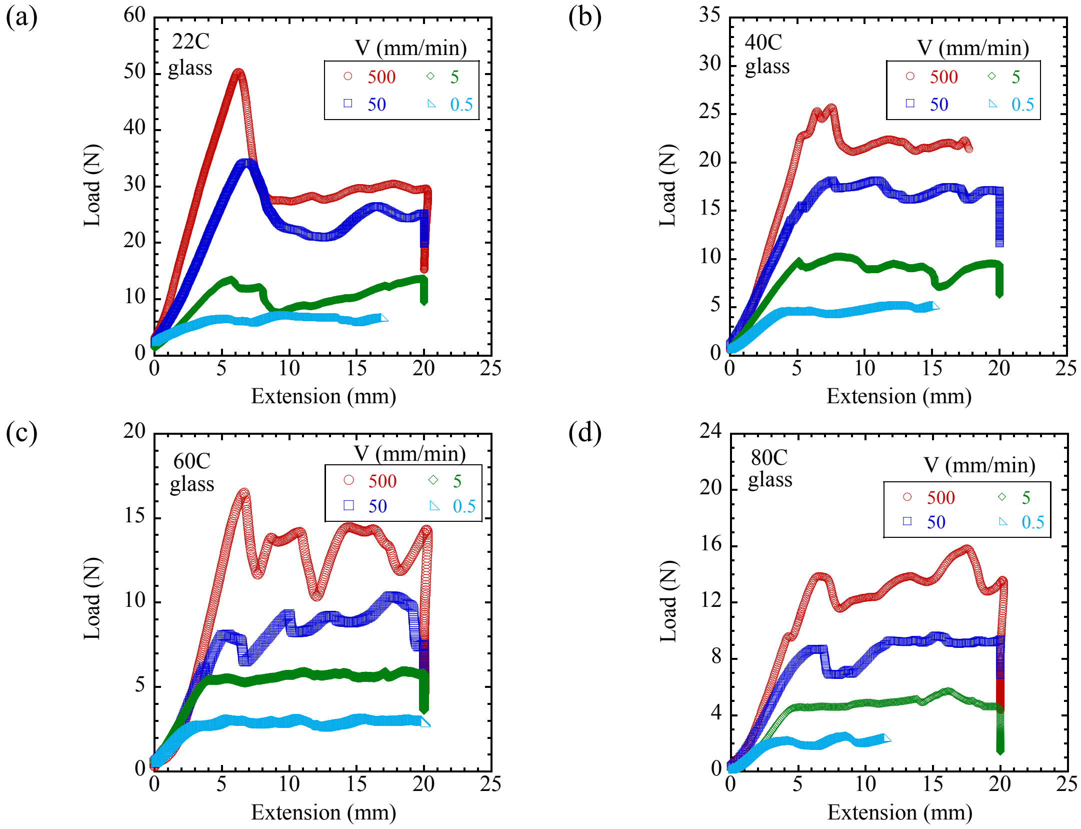

Figure SI.7a-d Peel test of VHB at different temperatures. Samples were adhered to glass for 5 minutes at 90C prior to experiment.

VIII. Relationship between P and Γ/P from peel test of VHB at different temperatures.

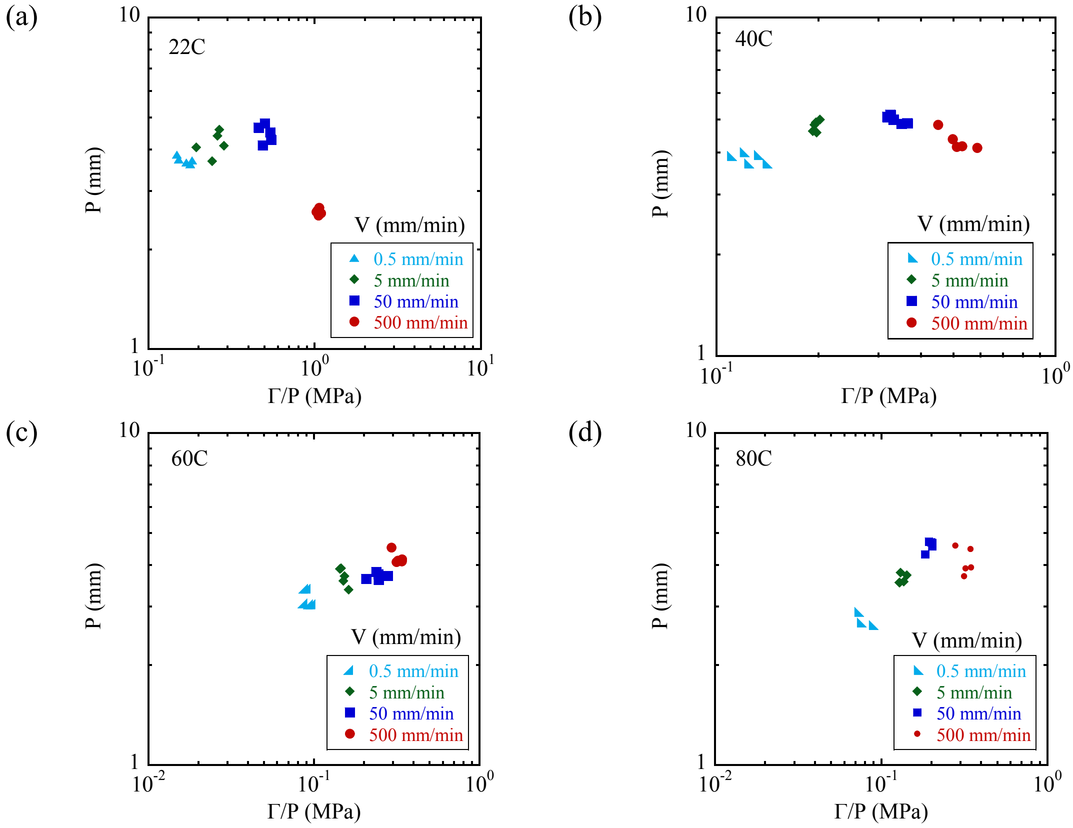

Figure SI.8a-d Relationship between P and Γ/P. The data points here were selected from five representative points within the steady-state region of each peeling curve shown in Fig. SI.7. The corresponding P values were then determined from video recordings of the experiments.

IX. Relationship between Γ/P and P/v_p from peel test of VHB at different temperatures.

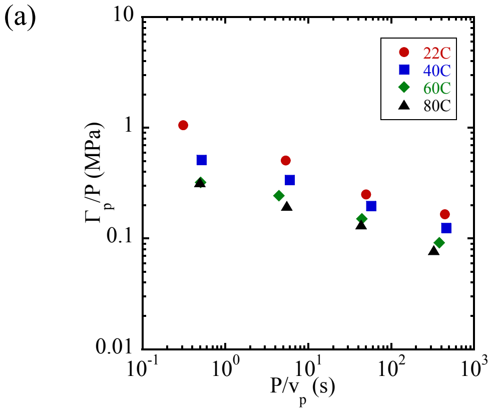

Figure SI.9 For each peeling rate–temperature condition, five data points were collected in Fig. SI.8, resulting in 16×5 total data points. The five points under each condition were averaged to obtain representative values, with P and Γ/P averaged separately. The averaged results are plotted in this figure.

X. Tensile adhesion tests on glass, metal, PI and Mylar substrates

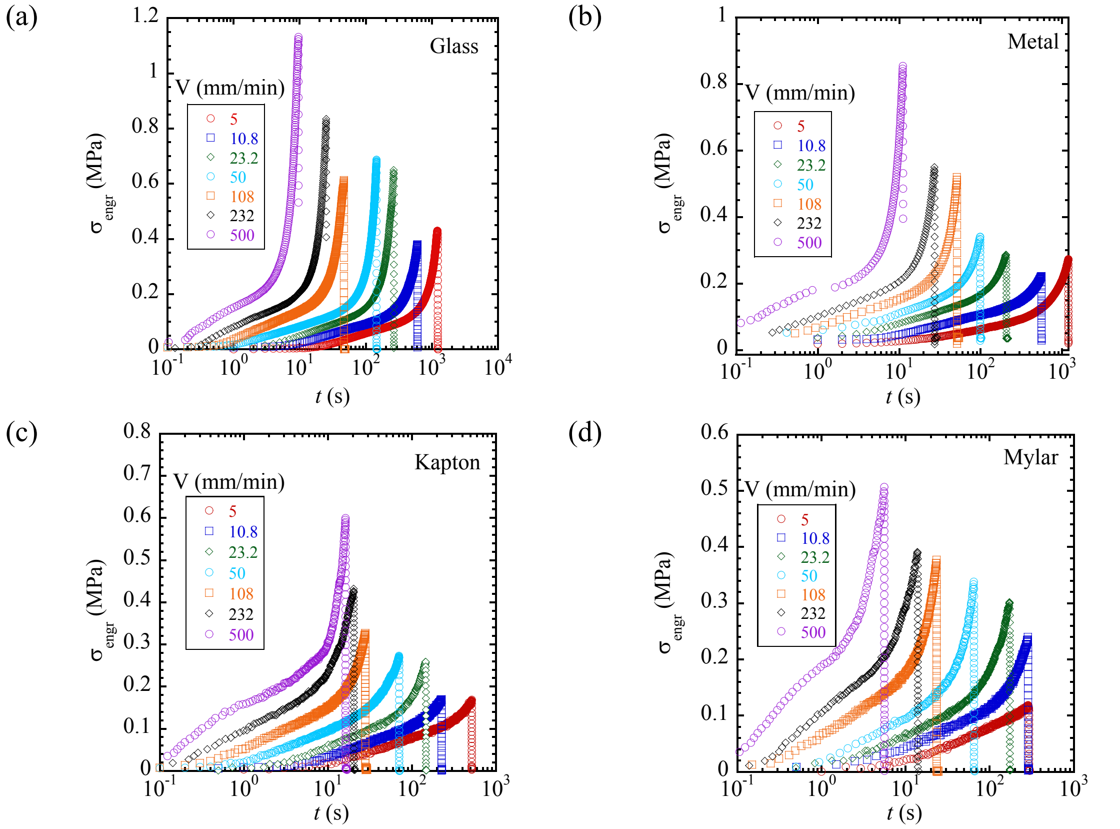

Figure SI.10 (a) Tensile adhesion test of VHB on glass. (b) Tensile adhesion test of VHB on metal. (c) Tensile adhesion test of VHB on Kapton film. (d) Tensile adhesion test of VHB on mylar. Samples were adhered to substrates for 5 minutes at 90C prior to experiment.

XI. Relationship between Γ/P and P/v_p from peel test of VHB on different substrates at different temperatures.

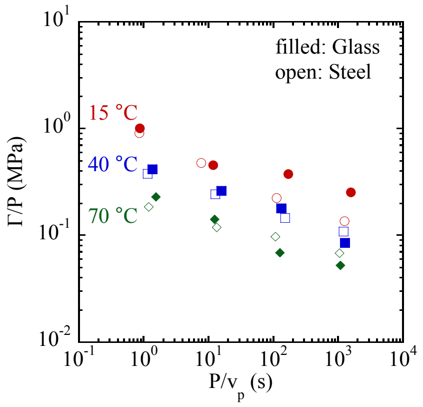

Figure SI.11 Relationship between Γ/P and P/v_p obtained from peel tests of VHB on different substrates at various temperatures. The data were analyzed following the same procedure as described in Fig.SI.6–SI.8. Filled symbols represent glass substrates, and open symbols represent metal substrates.
